# Cryptic speciation in the *Triatoma sordida* subcomplex (Hemiptera, Reduviidae) revealed by chromosomal markers

**DOI:** 10.1186/s13071-015-1109-6

**Published:** 2015-09-29

**Authors:** Francisco Panzera, Sebastián Pita, Julieta Nattero, Yanina Panzera, Cleber Galvão, Tamara Chavez, Antonieta Rojas De Arias, Lourdes Cardozo Téllez, François Noireau

**Affiliations:** Sección Genética Evolutiva, Facultad de Ciencias, Universidad de la República, Calle: Iguá 4225, 11400 Montevideo, Uruguay; Cátedra Introducción a la Biología, Facultad de Ciencias Exactas Físicas y Naturales, Instituto de Investigaciones Biológicas y Tecnológicas (IIByT) CONICET, Universidad Nacional de Córdoba, Córdoba, Argentina; Present address: Departamento de Ecología, Genética y Evolución, Laboratorio de Eco-Epidemiología, Facultad de Ciencias Exactas y Naturales, Universidad de Buenos Aires, Buenos Aires, Argentina; Laboratório Nacional e Internacional de Referência em Taxonomia de Triatomíneos (LNIRTT), Instituto Oswaldo, Cruz, Rio de Janeiro Brazil; Instituto Nacional de Laboratorios de Salud (INLASA), Laboratorio de Entomología Médica, La Paz, Bolivia; Centro para el Desarrollo de la Investigación Científica (CEDIC)/Díaz Gill Medicina Laboratorial/Fundación Moisés Bertoni, Asunción, Paraguay; Laboratorio de Biotecnología, Centro de Investigación Hernando Bertoni, Instituto Paraguayo de Tecnología Agraria, Asunción, Paraguay; Interactions hôtes-vecteurs-parasites dans les infections par trypanosomatidae (INTERTRYP), Institut de Recherche pour le Développement (IRD), Montpellier, France

**Keywords:** Sordida subcomplex species, Triatominae, Chagas disease vectors, Cryptic species, Holocentric chromosomes, FISH

## Abstract

**Background:**

Chagas disease vectors (Hemiptera-Reduviidae) comprise more than 140 blood-sucking insect species of the Triatominae subfamily. The largest genus is *Triatoma*, subdivided in several complexes and subcomplexes according to morphology, ecology and genetic features. One of them is the sordida subcomplex, involving four species: *Triatoma sordida, T. guasayana*, *T. garciabesi* and *T. patagonica*. Given the great morphological similarity of these species, their taxonomic identification, evolutionary relationships and population differentiation have been controversial for many years and even today remain under discussion.

**Methods:**

We simultaneously analyzed two chromosomal markers, C-heterochromatin distribution and 45S ribosomal genes chromosomal position, of 139 specimens from several sordida subcomplex populations from Argentina, Bolivia, Brazil and Paraguay, collected both in nature and from several established insectaries. Our results were compared with COI sequences deposited in GenBank.

**Results:**

We recognized five chromosomal taxa with putative hybrids, which each differ in at least one chromosome marker. Most of them present significant differences in their mtDNA sequences.

**Conclusion:**

The chromosomal taxa here show a significant chromosome differentiation involving changes in the C-heterochromatin content and in the ribosomal clusters position. This paper identifies several erroneously classified populations by morphological methods, delimits the geographical distribution of each taxon and proposes the existence of a new cryptic species, widely distributed in Argentina. We also suggest that sordida sibling species involve closely related as well as evolutionary distant species. Taxonomic status of each chromosomal taxon is discussed considering phenotypic and genetic results previously published.

## Background

The subfamily Triatominae (Hemiptera, Reduviidae) comprises more than 140 blood-sucking insect species – most of them vectors of the protozoan *Trypanosoma cruzi*, causative agent of Chagas disease or American trypanosomiasis. Of these, the most conspicuous genus is *Triatoma* with 80 species, grouped in 8 complexes and 8 subcomplexes according to morphology, habitat, ecology and genetic analyses [1]. One of these groups is the sordida subcomplex, which traditionally included four species: *Triatoma sordida* (Stål 1859)*, T. patagonica* Del Ponte 1929, *T. guasayana* Wygodzinsky & Abalos 1949 and *T. garciabesi* Carcavallo, Cichero, Martínez, Prosen & Ronderos 1967. *Triatoma sordida* was the first described species in 1859 and it presents the most extensive distribution in parts of central Argentina, Bolivia, Brazil, Paraguay, and Uruguay. *T. guasayana* is found in Argentina, Bolivia and Paraguay, while *T. garciabesi* and *T. patagonica* are recorded only from Argentina [2, 3].

The taxonomic validity of sordida subcomplex species, their evolutionary relationships, and especially population differentiation within *T. sordida*, have been controversial for many years and even today remain under discussion.

The first report regarding morphological variation among *T. sordida* populations appeared in 1951 [4]. These authors compared smaller and darker sylvatic individuals from Santiago del Estero (northeastern Argentina) with domestic and peridomestic specimens from the rest of Argentina. In 1965, electrophoretic profiles of hemolymph proteins showed significant differentiation among Argentinean sylvatic samples with domestic specimens from Brazil [5]. These sylvatic specimens were then described as a new species called *T. garciabesi* based on morphological differences from *T. sordida* and *T. guasayana* [6], but this new species was then synonymized with *T. sordida* [2]. Several years later, isoenzymatic and chromosomal studies of several *T. sordida* populations from Argentina and Brazil suggested the existence of at least two distinct forms, one from Brazil and other from Argentina [7]. Based on these results, *T. garciabesi* was revalidated as a species, according to differences in their morphology (overall size, color, head and genitalia), isoenzymes (10 diagnostic loci) and chromosomal characteristics (C-heterochromatin distribution) [8]. In Bolivia, isoenzymes studies suggested the existence of two distinct forms within the *T. sordida* populations, named group 1 and group 2, differentiated by their Idh-2 and Mdh-2 loci [9]. All domestic *T. sordida* populations in Bolivia were of group 1, although this form could also be found in peridomestic and sylvatic environments [10]. *T. sordida* group 2 is predominantly sylvatic and seems to be restricted to the Chaco region. In the Bolivian Chaco, both *T. sordida* groups and *T. guasayana* occurred in sympatry, including putative hybrids [9–11]. Morphometric (wings and heads) and cuticular hydrocarbon analyses confirmed a distinction between *T. garciabesi* and *T. sordida*, and also show significant differences between *T. sordida* populations from Argentina and Brazil [12, 13]. More recently, population studies on *T. sordida* from Paraguay revealed ecological, genetic and morphometric differences between specimens from Western (Chaco region) and Eastern Paraguay [14].

Karyotypic information for more than 80 species of Triatominae is currently available, showing a highly conserved diploid chromosome number ranging from 21 to 25 chromosomes in males [15]. In spite of this homogeneity, cytogenetic analyses have shown that the subfamily is one of the most variable and chromosomally diverse of the Heteroptera. Distribution, size and amount of C-heterochromatin and the chromosomal location of 45S ribosomal genes presented a striking differentiation among triatomine species. Although there are exceptionally polymorphic species, both traits are species-specific characters, and therefore very useful for species discrimination [15–18].

The four sordida subcomplex species exhibit the same diploid number: 22 chromosomes composed of 20 autosomes plus XY/XX (male/female) sex chromosomes [7, 8]. The original *T. sordida* karyotypic information was described for Brazilian specimens [19]. With C-banding, *T. guasayana* and *T. garciabesi* did not present autosomal C-heterochromatin while *T. sordida* (Brazilian populations) and *T. patagonica* presented C-blocks comprising around the 30 % of their autosomes [7]. Currently, Fluorescence in situ hybridization (FISH) analyses are restricted to one *T. sordida* population from Brazil and one *T. garciabesi* population. In both species 45S ribosomal DNA clusters are located on X chromosome [17].

In the current work, we analyzed both chromosomal markers simultaneously on specimens from Argentina, Bolivia, Brazil and Paraguay. This study does not include *T. patagonica* because it does not present morphological identification problems with the other species of the subcomplex [20]. The application of two cytogenetic techniques offers the possibility of differentiate populations that show similar characteristics with a single chromosomal marker, and the analysis with both markers allows us to detect the existence of hybrids, either between species or populations chromosomally differentiated, as recently reported in *T. infestans* [16]. Both markers are mainly inherited in a Mendelian fashion and evolved independently [16, 17, 21], so they are suitable for analyzing different evolutionary pathways.

## Methods

### Insects and sampling sites

We carried out chromosomal analyses of 139 insects of the *T. sordida* subcomplex from Argentina, Bolivia, Brazil and Paraguay, including field-caught and insectary specimens. The geographic origin of each population, habitat, and *a priori* species determination are given in Tables 1, 2, 3, 4 and 5. Some individuals examined cytogenetically in this paper have been previously studied using various morphological and genetic analyses (Tables 1–5). When possible, we determined the chromosomal characteristics of each species based on topotype specimens (i.e. individuals from the same geographical origin as the holotype). For *T. sordida,* named here *T. sordida sensu stricto,* we considered as topotypes the specimens from Minas Gerais, Brazil, because the holotype locality is not specified. For *T. garciabesi* we analyzed the same individuals used in their redescription [8] and chromosomal characteristics were also determined from specimens from the holotype locality (Santiago del Estero, Argentina) and from the same specimens previously identified by isoenzymes [7, 9]. A new chromosomal taxon, here referred to as *T. sordida Argentina*, was differentiated for specimens from San Luis del Palmar (Corrientes, Argentina), and a further chromosomal taxon was differentiated from domestic populations from La Paz (Bolivia) provisionally named *T. sordida La Paz.*

**Table 1:** Populations identified as *T. guasayana* by C-banding and rDNA FISH studies

Country, Province, Department, habitat	*N*	Original species assignment	Previous morphological and genetic analyses
Argentina, Santiago del Estero, P. CRV. 4^th^gener.	3	*T. guasayana*	Cuticular hydrocarbons, iso-enzymes, C-banding [7, 13, 39].
Argentina, Córdoba, Cruz del Eje, S. CRV. 2^th^gener.	5	*T. guasayana*	Isoenzymes, C-banding [7].
Argentina, Córdoba, Sobremonte, S.	3	*T. guasayana*	NS
Argentina, La Rioja, P. CRV. Colony 0297, 4^th^gener.	4	*T. guasayana*	Isoenzymes, C-banding [7].
Bolivia, Santa Cruz, Boyuibe, S.	2	*T. guasayana*	Isoenzymes [9].
Bolivia, Santa Cruz, Izozog, S.	4	*T. guasayana*	Isoenzymes [9, 10].
Bolivia, Cochabamba, Mataral, S.	1	*T. guasayana*	NS
Bolivia, Cochabamba, Chujillas. S. LEN. 2^th^gener.	2	*T. guasayana*	NS

Geographical origin, number of analyzed individuals and previous reports

*N* number of analyzed individuals in this paper, *CRV* Centro de Referencia de Vectores, Servicio Nacional de Chagas (Córdoba, Argentina), *LNIRTT* Laboratório Nacional e Internacional de Referencia em Taxonomia de Triatomíneos, Instituto Oswaldo Cruz (Fiocruz) (Rio de Janeiro, Brazil), *LEN* Laboratorio de Entomología Médica, Instituto Nacional de Laboratorios de Salud (INLASA) (La Paz, Bolivia), *D* domestic, *P* peridomestic, *S* sylvatic, *gener* generation, *RAPD* Random Amplified Polymorphic DNA, *NS* No Studied

**Table 2:** Populations identified as *T. garciabesi* by C-banding and rDNA FISH studies

Country, Province, Department, habitat	*N*	Original species assignment	Previous morphological and genetic analyses
Argentina, Salta, Rivadavia, S. LNIRTT.	6	*T. garciabesi*	Morphology of head and genitalia, isoenzymes, C-banding, FISH [8, 17].
Argentina, La Rioja, P. CRV. Colony 0303, 3^th^gener.	2	*T. sordida/T. garciabesi*	Geometric morphology of wings and heads, isoenzymes, C-banding [7, 12].
Argentina, Santiago del Estero, Rio Hondo, S.CRV. 4^th^ gener.	2	*T. sordida/T. garciabesi*	Cuticular hydrocarbons, isoenzymes, C-banding [7, 13].
Argentina, Santiago del Estero, Aguirre, P.	3	*T. garciabesi*	NS
Argentina, Chaco, Guemes, P. CRV. Colony 352, 1^th^gener.	5	*T. sordida*	NS
Argentina, Formosa, Patiño, P. CRV. Colony 335, 1^th^gener.	3	*T. sordida*	NS
Paraguay, Presidente Hayes & Boquerón. Several localities. P.	9	*T. sordida*	Geometric morphology of wings and heads, RAPD [14].
Bolivia, Santa Cruz, Izozog. S.	9	*T. sordida Group 2*	Isoenzymes [9, 10].

Geographical origin, number of analyzed individuals and previous reports. Abbreviations described in Table 1

**Table 3:** Populations identified as *T. sordida sensu stricto* by C-banding and rDNA FISH studies

Country, Province, Department, habitat	*N*	Original species assignment	Previous morphological and genetic analyses
Brazil, Minas Gerais, Montes Claros, P. LNIRTT. 1^th^-2^th^gener.	8	*T. sordida*	Morphology of head and genitalia, isoenzymes, C-banding [7, 8].
Brazil, Minas Gerais, Uberaba. P.	4	*T. sordida*	Isoenzymes, C-banding [7].
Brazil, Piaui, P. LNIRTT.	7	*T. sordida*	Isoenzymes, C-banding [7].
Brazil, Matto Grosso, São José do Povo, P.	2	*T. sordida*	C-banding, FISH [17].
Bolivia, Santa Cruz, Izozog. P.	2	*T. sordida Group 1*	Isoenzymes [9, 10].
Bolivia, Santa Cruz, Cotoca. D.	2	*T. sordida Group 1*	Isoenzymes [9, 10].
Paraguay, San Pedro, P.	2	*T. sordida*	Geometric morphology of wings and heads, RAPD [14].
Paraguay, Concepción, P.	7	*T. sordida*	NS

Geographical origin, number of analyzed individuals and previous reports. Abbreviations described in Table 1

**Table 4:** Populations identified as *T. sordida Argentina* by C-banding and rDNA FISH studies

Country, Province, Department, habitat	*N*	Original species assignment	Previous morphological and genetic analyses
Argentina, Corrientes, San Luis del Palmar. P.	4	*T. sordida*	Geometric morphology of wings and heads [12].
Argentina, Santiago del Estero. S. LNIRTT.	2	*T. sordida*	NS
Argentina, Chaco, Guemes, El Colchón, P.	6	*T. sordida*	NS
Argentina, Formosa, Pirane, P. CRV. 1^th^ gener.	2	*T. sordida*	NS
Argentina, Tucumán, San Miguel, D.	3	*T. sordida*	NS
Bolivia, Cochabamba, Quilacollo, Cotapachi, S.	3	*T. sordida*	NS
Paraguay, Paraguari, Carepaguá, P.	6	*T. sordida*	Geometric morphology of wings and heads, RAPD [14].
Paraguay, San Pedro, Itacurubí& Villa Rosario, P.	3	*T. sordida*	Geometric morphology of wings and heads, RAPD [14].

Geographical origin, number of analyzed individuals and previous reports. Abbreviations described in Table 1

**Table 5:** Populations identified as *T. sordida La Paz* by C-banding and rDNA FISH studies

Country, Province, Department, habitat	*N*	Original species assignment	Previous morphological and genetic analyses
Bolivia, La Paz, Inquisivi, D	4	*T. sordida*	NS
Bolivia, La Paz, Apolo, D	5	*T. sordida*	NS

Geographical origin, number of analyzed individuals and previous reports. Abbreviations described in Table 1

### Chromosome preparations and banding procedures

For chromosome preparations, testes were removed from freshly killed adults, and fixed in an ethanol–glacial acetic acid mixture (3:1,v:v). Subsequently, we performed C-banding to establish the diploid chromosome number (2n) and the C-heterochromatin distribution [21]. We also applied FISH analyses to determine the location of 45S ribosomal clusters [17]. For each specimen, at least 20 cells were analyzed. Chromosome preparations were examined under a Nikon Eclipse 80i microscope and the images were obtained with a DS-5Mc-U2 digital camera using Nikon Nis Element s3.1 Advanced Research software and processed with Adobe Photoshop® software.

### Molecular datasets, sequence alignments and phylogenetic analyses

Fourteen *T. sordida, T. garciabesi* and *T. guasayana* partial COI gene sequences deposited in GenBank were employed for phylogenetic analyses, plus two *T. rubrovaria* sequences used as outgroup. Other sequences of this subcomplex available in GenBank were excluded from this study since their geographical origin was unknown. All sequence accession numbers are specified in Fig. 3. Maximum likelihood (ML) phylogenetic analyses were implemented in MEGA 6 [22] with statistical support for the nodes evaluated with 1000 bootstrap replicates. The best fitted substitution model was also determined using MEGA 6 software [22]. The alignment included 522 bp including 113 variable sites (21.6 %), 101 of which were informative for parsimony (19.3 %).

## Results

Male specimens from all populations showed the same diploid number (2n = 22) constituted by 20 autosomes plus two sex chromosomes (XY). All individuals, including putative hybrids, presented normal meiosis without irregularities in the chromosome behavior. With C-banding, all specimens had a C-heterochromatic Y chromosome. Different C-banding patterns are observed according to the number of autosomes with C-regions. The ribosomal cluster has one or two chromosome loci per haploid genome, showing 4 location patterns: one autosomal pair, X chromosome, both sex chromosomes or on an autosomal pair and one sex chromosome simultaneously.

Analysis of each individual with both chromosomal markers allowed us to identify five different chromosomal taxa, which are described below. Tables 1–5 show the populations identified within each taxon. Table 6 summarizes chromosomal characteristics of the five chromosomal taxa identified.

**Table 6:** Comparative summary of the 5 chromosomal taxa and putative hybrids identified by two chromosomal markers

Chromosomal taxon (*n*)	Autosomal C-heterochromatin by C-banding	Location of 45S ribosomal clusters by FISH
*T. guasayana* (24)	NO	One autosomal pair
*T. garciabesi* (39)	NO	X chromosome
*T. sordida sensu stricto* (34)	YES	X chromosome
*T. sordida Argentina* (29)	YES	X and Y chromosomes
*T. sordida La Paz* (9*)*	YES	One autosomal pair
Hybrids from Apolo-La Paz (2) and Izozog-Santa Cruz (2)	YES	Polymorphic: One autosomal pair plus X chromosome or on a heterozygote autosomal pair.

We included the total number of analyzed individuals between brackets

**Fig. 1:**
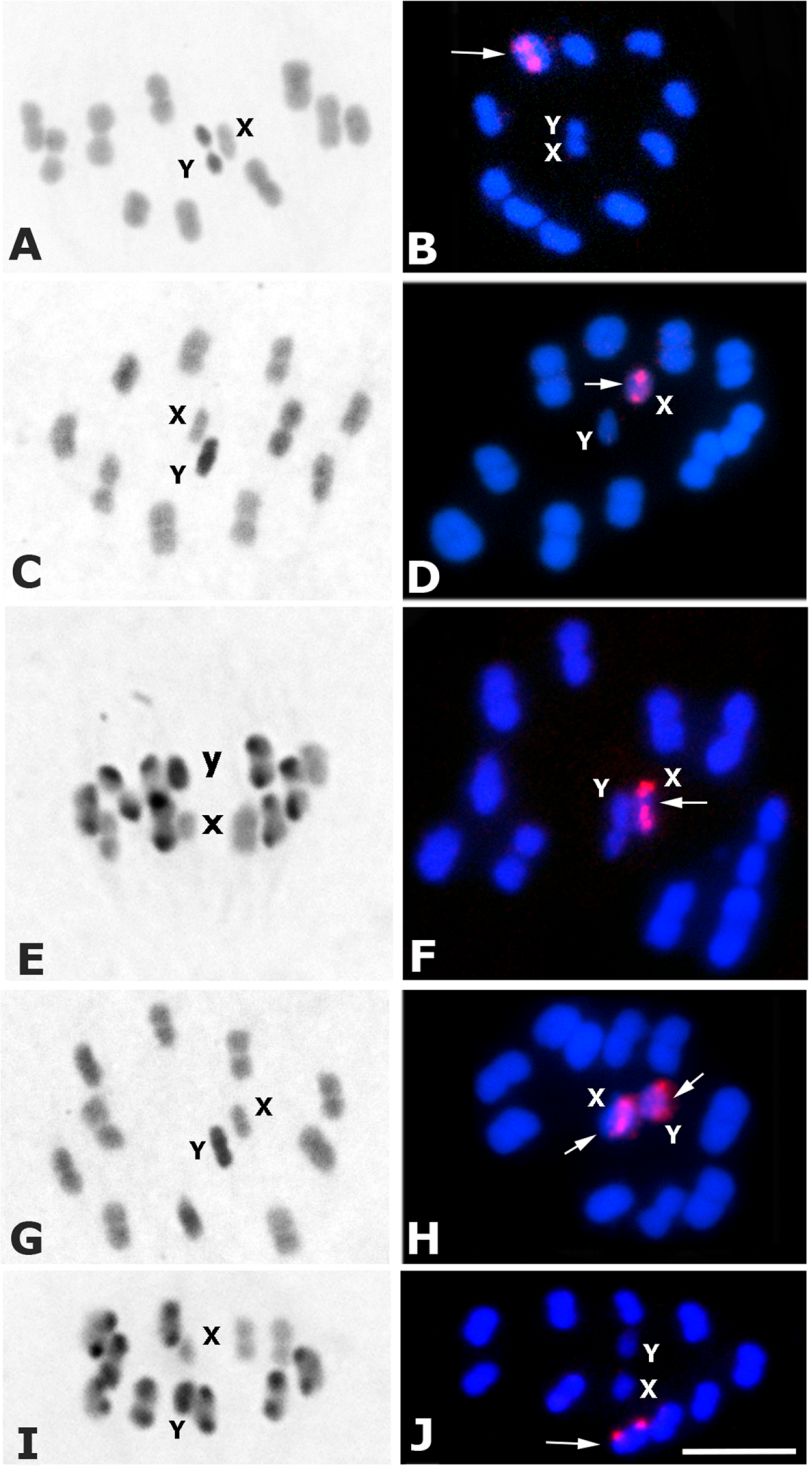
Male meiosis in different *Triatoma sordida* subcomplex species (2n = 20A + XY). (**a**-**c**-**e**-**g**-**i**): C-banding. (**b**-**d**-**f**-**h**-**j**): Fluorescent in situ hybridization with 45S ribosomal DNA probe. **a**-**b**: *T. guasayana* **a**: Metaphase I (MI). All autosomal bivalents and the X chromosome are euchromatic while that the Y appears C-heterochromatic. **b**: Metaphase II (MII). rDNA signals are located in one autosomal bivalent (*arrow*). **c**-**d**: *T. garciabesi* **c**: MI. C-heterochromatin distribution similar as observed in *T. guasayana* (**d**): M I. Ribosomal signals on the X chromosome (*arrow*). **e**-**f**: *T. sordida sensu stricto.* **e**: MII. Seven autosomal pairs exhibit C-blocks while 3 pairs are euchromatic (**f**): MI. rDNA signals on the X chromosome (*arrow*). **g**-**h**: *T. sordida Argentina.* **g**: MI. All chromosome complement is euchromatic, except for the heterochromatic Y chromosome. **h**: MI. Ribosomal signals on both sex chromosomes (*arrows*). **i**-**j**: *T. sordida La Paz*. **i**: MII. Eight autosomal pairs present C-blocks, while 2 pairs are euchromatic. **j**: MII. rDNA clusters on one autosomal pair (*arrow*). Bar = 10 μm

***T. guasayana*** (Table 1 and Fig. 1a, b): The 10 autosomal pairs do not show striking size differences, being two or three pairs slightly larger. The X chromosome and all bivalents have no C-bands (Fig. 1a). Ribosomal clusters are located in one autosomal pair (arrow Fig. 1b). This species included samples from Argentina and Bolivia. In several individuals our chromosomal identification matches the species assignment by isoenzymes (Table 1).

***T. garciabesi*** (Table 2 and Fig. 1c, d): The 10 bivalents exhibit similar size, but two or three of them slightly larger. Autosomes and X chromosome are euchromatic (without C-bands) (Fig. 1c). The 45S rDNA clusters are localized on the X chromosome (arrow Fig. 1d). This species included specimens from Argentina, Western Paraguay and Bolivian Chaco.

***T. sordida sensu stricto*** (Table 3 and Fig. 1e, f): Two or three autosomal pairs are slightly larger than the rest. Most autosomal pairs present a C-heterochromatic block in one chromosomal end, while the others are euchromatic (Fig. 1e). The X chromosome may present a small C-block or not. The 45S rDNA clusters are localized on the X chromosome (arrow Fig. 1f). This species included samples from Brazil, Central and Eastern Paraguay, and Bolivian Chaco.

***T. sordida Argentina*** (Table 4 and Fig. 1g, h): The 10 autosomal pairs and the X chromosome do not have C-heterochromatin (Fig. 1g). Ribosomal clusters are located on both sex chromosomes (X and Y) (arrows Fig. 1h). This putative chromosomal species included samples from Argentina, Eastern Paraguay, and Bolivian high valleys (Table 4).

***T. sordida La Paz*** (Table 5 and Fig. 1i, j): Seven to eight autosomal pairs present a C-heterochromatic region in only one chromosomal end, similar to that observed in *T. sordida sensu stricto* (Fig. 1i). However, the ribosomal clusters are located on one autosomal pair (arrow Fig. 1j). This putative chromosomal species included exclusively domestic individuals from Bolivian highlands of La Paz (Table 5).

**Fig. 2:**
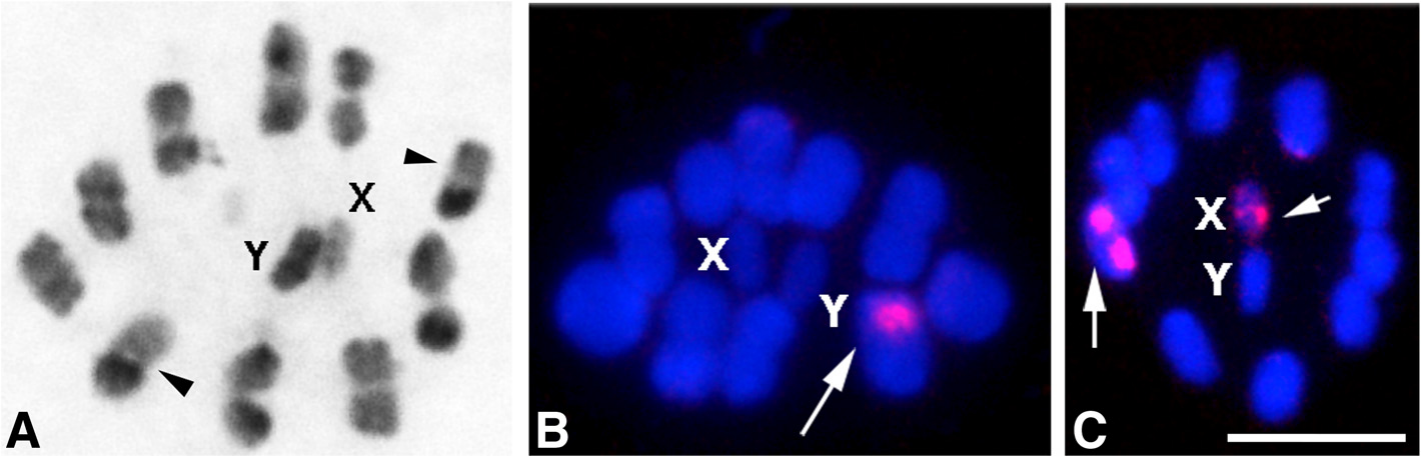
Male meiosis in putative hybrids of *Triatoma sordida* subcomplex (2n = 20A + XY). **a**: Metaphase I with C-banding. Three types of bivalents are observed: heterochromatic (C-region in one chromosomal end), euchromatic (without C-region) and heterozygote bivalents with one homologous heterochromatic and the other euchromatic (*arrowheads*). **b**-**c**: Metaphase I and metaphase II, respectively. Fluorescent *in situ* hybridization with 45S ribosomal DNA probe. Ribosomal signals (*arrows*) can be located in one heterozygote autosomal pair (**b**) or in an autosomal pair and X chromosome simultaneously (**c**). Bar = 10 μm

***Chromosomal hybrids*** (Fig. 2a, c): We detected 4 individuals from Bolivia (two from Apolo-La Paz and two from Izozog-Santa Cruz) with chromosomal patterns that could represent hybrids between some of the above mentioned taxa. With C-banding, each individual presented three types of bivalents: heterochromatic with C-region in one chromosomal end, euchromatic and heterozygote bivalents (one homologous heterochromatic and the other euchromatic) (arrowheads Fig. 2a). With FISH technique, they exhibit two different ribosomal cluster locations: either in one heterozygote autosomal pair, or in an autosomal pair plus X-chromosome (arrows Fig. 2b, c, respectively).

### Phylogenetic analyses

**Fig. 3:**
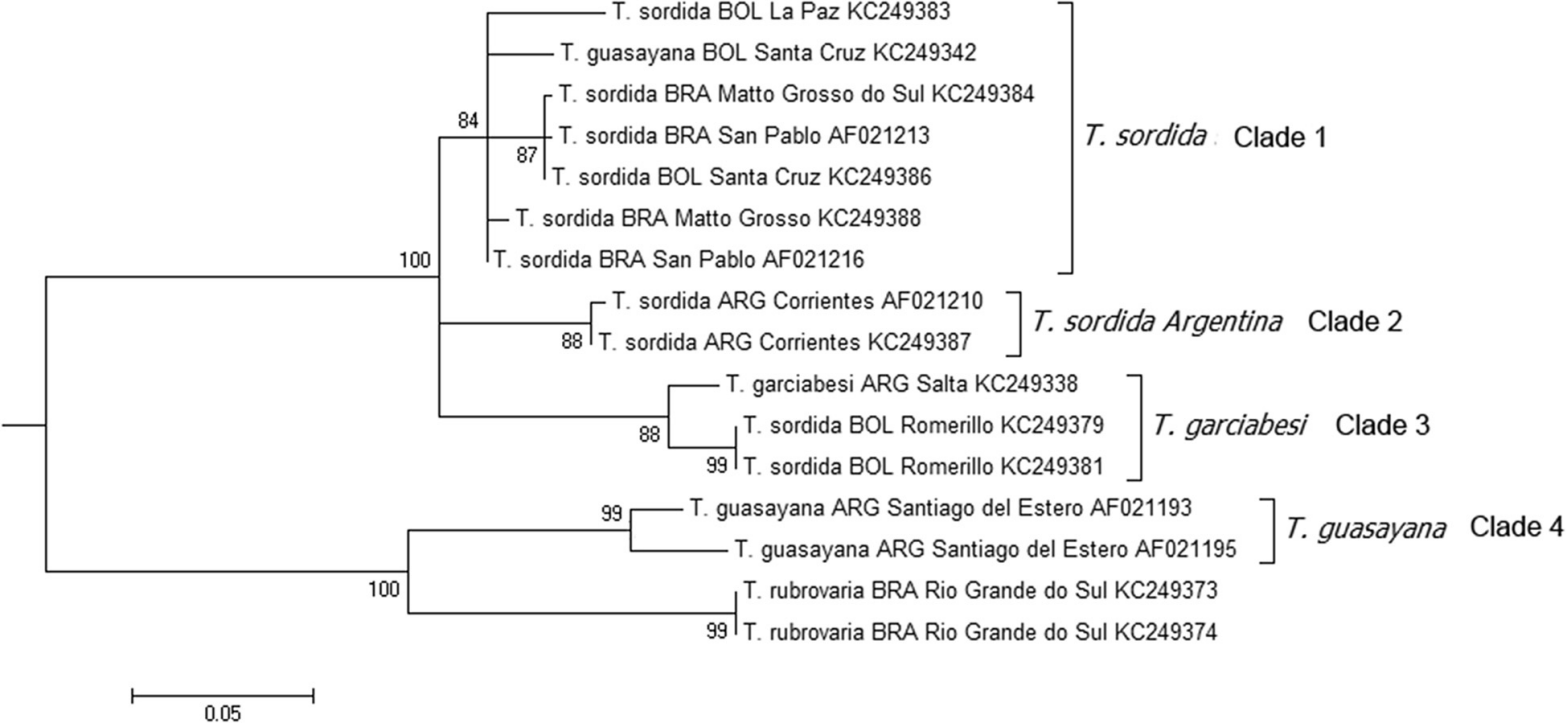
Maximum likelihood phylogenetic tree obtained with COI gene partial sequences deposited in GenBank. This tree clearly shows four well supported clades and mistakes in the primary species identification of some individuals (KC249342, KC249379 and KC249381). Numbers on nodes represent statistical support obtained through 1000 bootstrap replications. ARG = Argentina; BOL = Bolivia; BRA = Brazil

The maximum likelihood tree was obtained using Tamura 3-parameters plus Gamma distribution (T92 + G). The resulting ML tree topology (Fig. 3) showed four well supported groups or clades. Clade 1 or *T. sordida*: include 6 *T. sordida* samples (4 from Brazil and 2 from Bolivia) and 1 specimen identified as *T. guasayana* (Bolivia); Clade 2 or *T. sordida Argentina*: include 2 samples identified as *T. sordida* from Corrientes (Argentina); Clade 3 or *T. garciabesi*: Include 1 *T. garciabesi* individual from Argentina (Salta) and 2 specimens identified as *T. sordida* from Bolivian Chaco (Romerillo); Clade 4 or *T. guasayana:* involve 2 *T. guasayana* individuals from Argentina (Santiago del Estero).

The mean pairwise nucleotide distances (Tamura-3 parameters) between the four clades reflect significant differences between them (from 5.3 to 14.9 %) (Table 7). Considering the robustness of the four clades supported by their genetic distance, we can suggest errors in the original taxonomic determination of some individuals (*T. guasayana*/KC249342; *T. sordida* from Romerillo/KC249379 and KC249381), since they grouped within a clade formed by individuals from another species (Fig. 3). These misidentifications were corroborated by our cytogenetic results (see discussion) using individuals from the same or similar geographic locations.

**Table 7:** Mean Tamura-3 parameters pairwise genetic distances between the four clades for the COI gene fragments

	CLADE 1	CLADE 2	CLADE 3	CLADE 4	*OUTGROUP*
	*T. sordida*	*T. sordida Argentina*	*T. garciabesi*	*T. guasayana*	*T. rubrovaria*
CLADE 1	**1.4**				
CLADE 2	5.3	**0.4**			
CLADE 3	7.1	7.0	**2.0**		
CLADE 4	14.9	14.6	14.0	**3.6**	
OUTGROUP	15.7	13.5	14.6	10.3	**0.0**

Intergroup distances are in the lower left section; mean intragroup distances are in bold

## Discussion

Cryptic species, also called sibling or isomorphic species, are identical in their external appearance, or differ in apparently minor and not easily visible traits. In Triatominae, the use of different phenotypic and genetic markers has shown that cryptic speciation is a widespread phenomenon in this subfamily. The existence of sibling species has been described in different *Triatoma* groups such as the brasiliensis, dimidiata, phyllosoma and sordida subcomplexes, as well as in several species of *Rhodnius* and *Panstrongylus* (for review see [1]).

Sordida subcomplex species except *T. patagonica* are morphologically very similar, with overlapping geographical distribution and even sympatric in many regions with putative hybrids, which significantly increase the taxonomic confusion. Although there are phenotypic and genetic markers able to differentiate the species, its recognition requires high expertise which greatly hinders their application by vector control staff– leading to mistaken determination even within insectary material (see Tables 1–4). Below, we discuss our results for each chromosomal taxon here identified, in comparison with previously published determinations.

***T. guasayana***: The morphological differentiation of *T. guasayana* with *T. sordida* is very difficult, especially in the nymphal stages. The geographic distribution of both species overlaps in northern Argentina and in the Chaco region from Bolivia and Paraguay. *T. guasayana* is mainly sylvatic, occupying a great variety of habitats including bromeliads, similar to *T. sordida*. Peridomiciliary colonies are frequent, side by side with *T. infestans* and *T. sordida*, especially in chicken houses [2]. Isoenzymes studies in Bolivia show the absence of hybrid forms confirming the reproductive isolation in nature of both species [10].

Genetic markers (isoenzymes, chromosomal and mitochondrial sequences) clearly differentiated *T. guasayana* from the other species of sordida subcomplex [7, 9, 10, 23–25]. However, probably due to their morphological similarity, COI sequences deposited in GenBank reveal at least one mistaken identification: a specimen from Santa Cruz, Bolivia (Chaco, Tita) (KC249342) originally identified as *T. guasayana*, should be recognized as *T. sordida* (Fig. 3). At phylogenetic level, genetic distances between *T. guasayana* and the other sordida taxa are the most extreme, between 14 and 14.9 % (Table 7). In fact *T. guasayana* seems more related to *T. rubrovaria* (10.3 %) (Table 7), as suggested by other authors using different mitochondrial genes [23–25]. Chromosomal similarities between these two species (lack of autosomal heterochromatin and ribosomal clusters on one autosomal pair) would also support the inclusion of *T. guasayana* in the rubrovaria subcomplex.

***T. garciabesi***: Until now, this species can be only described in Central and Northern Argentina [8, 12, 13]. Our results extend the *T. garciabesi* geographical distribution to other Chaco regions previously not described: Bolivian Chaco (Santa Cruz), western Paraguay (Departments of Boquerón and Presidente Hayes) and the Argentine provinces of Tucumán and Santiago del Estero (Table 2). This geographical distribution closely matches the predicted distribution for *T. garciabesi* based on ecological niche modeling [12]. Most of them are sylvatic, but occasionally occupy peridomestic environments.

Different approaches support the taxonomic validity of this species: head and genitalia morphology [8], head and wing morphometrics [12], cuticular hydrocarbons [13], isoenzymatic and cytogenetic traits [7, 17], and molecular analyses [26] (Fig. 3). This paper establishes the chromosomal identity between *T. garciabesi* and the *T. sordida* group 2 from Bolivia defined by isoenzymes [9, 10].

Despite the genetic and phenotypic differentiation, taxonomic problems still persist in terms of distinguishing *T. garciabesi* from other species of the subcomplex. Individuals from established laboratory colonies (Salta, La Rioja, Santiago del Estero, Formosa and Chaco) which were originally identified as *T. sordida,* presented the chromosomal characteristics of *T. garciabesi* (Table 2). Hence, incorrect identification of this species is also seen in the sequences deposited in GenBank. The two individuals from the Bolivian Chaco (Romerillo) probably belong to *T. garciabesi* rather than *T. sordida* (Fig. 3). Recently, comparative analyses of *T. sordida* populations from Western (Chaco) and Eastern regions of Paraguay reveal striking differences in the feeding patterns, random amplified polymorphic DNA profiles (RAPD), and head and wing morphometrics [14]. These authors suggested that this differentiation is associated to eco-geographical isolation by distance. However, our chromosomal studies suggest that *T. sordida* populations in Paraguay involve at least three taxa. In the Chaco region (Western Paraguay) we only found *T. garciabesi*, while in the Eastern region it appears that *T. sordida Argentina* and *T. sordida sensu stricto* coexist in sympatry. The ecological differentiation and distinct feeding patterns described by [14] support our results. We suspect that besides *T. garciabesi* and *T. guasayana* [27], also other cryptic sordida species may be present in Paraguay.

***T. sordida sensu stricto***: This species is the most geographically widespread species of the sordida subcomplex, found in large parts of Argentina, Brazil, Bolivia (Santa Cruz) and Paraguay (Central and Eastern) [2]. Surprisingly according to our results, this species was not detected in Argentina despite the large number of populations and individuals analyzed (Tables 2–4). Individuals recognized as *T. sordida* group 1 in Bolivia by isoenzymes [9–11] belong to this chromosomal group (Table 3). All these populations occupy domestic and peridomestic habitats, although they are also found in sylvatic habitats such as birdnests, tree holes and under dry tree bark [9–11]. Historically, *T. sordida* is forming abundant colonies in peridomestic habitats (particularly chicken coops), with the ability to invade and colonize human habitations in Brazil [28, 29], Bolivia [9–11, 30] and Paraguay [14]. Considering the sibling taxa here analyzed, *T. sordida sensu stricto* would be the most significant in terms of Chagas disease transmission [31], being the most common synanthropic species captured in Brazil [28].

***T. sordida Argentina***: For this chromosomal taxon, we considered as topotypes the individuals from San Luis del Palmar (Corrientes, Argentina). Chromosomal characteristics (without autosomal C-heterochromatin and ribosomal clusters in both sex chromosomes) were determined in most individuals from Argentina and some from Bolivia and Paraguay, all of them initially identified as *T. sordida* (Table 4). Ribosomal genes located on both sex chromosomes (Fig. 1g, h) is an uncommon feature in the genus *Triatoma*, only previously observed in four unrelated species of the 27 so far analyzed [17]. The mtDNA sequence divergence between *T. sordida Argentina* with sympatric *T. garciabesi* is 7.0 % (Table 7), similar to that used to support specific denominations in the brasiliensis subcomplex [32] or *Mepraia* species [33]. Sequence divergence between *T. sordida Argentina* and *T. sordida sensu stricto* is 5.3 %, similar to that observed between *T. sanguisuga* subspecies [34]. However, isoenzymatic analyses involving 19 loci revealed a striking differentiation between *T. sordida Argentina* and *T. sordida sensu stricto* from Brazil, showing 3 different fixed alleles and 4 polymorphic loci [7]. Considering this isoenzymatic diversity and the extreme chromosomal distinction (C-heterochromatin amount and ribosomal clusters location) between these taxa we suggest there may be complete genetic isolation between them. We therefore propose that *T. sordida Argentina* presents characteristics consistent with its designation as a new species. At the epidemiological level, *T. sordida Argentina* were found in various sylvatic ecotopes and peridomestic habitats, but in very low frequency in domestic environments compared to, say, *T. sordida* from Brazil [35, 36].

***T. sordida La Paz***: This chromosomal taxon was identified in domestic specimens collected from La Paz (Bolivia) (Table 5). These individuals exhibit heterochromatic autosomes similar to what is observed in *T. sordida sensu stricto*, but they differ in the position of the ribosomal clusters (Fig. 1). *T. sordida La Paz* showed rDNA clusters in one autosomal pair, while *T. sordida sensu stricto* has them on the X chromosome. Considering almost 100 heteropteran species analyzed to date, including 40 triatomine species, the chromosomal position of ribosomal genes appears to be a species-specific character, although variation in ribosomal gene location was reported in *T. infestans* [16, 17]. For this reason we cannot rule out that *T. sordida sensu stricto* and *T. sordida La Paz* are conspecific populations with different ribosomal gene locations. Furthermore, some hybrid individuals detected in Bolivia (Fig. 2) could plausibly have originated from crosses between these two chromosomal taxa, thereby strengthening the idea of an intraspecific variation in ribosomal genes. Unfortunately we do not have information about COI sequences of individuals from this chromosomal taxon.

### Putative hybrids by chromosomal markers

Experimental crosses between *T. sordida* populations and with *T. guasayana* have been made by many researchers, showing either fertility or F1 sterility [8, 20, 37–39]. These results, apparently contradictory, can now be explained by the fact that the crossings involved distinct chromosomal taxa, as suggested in this paper. Currently, there is only one report that demonstrates the existence of natural hybrids in sordida subcomplex species. By isoenzymes, putative hybrids in low frequency (3 %) were recorded in two localities from Santa Cruz in Bolivia (Izozog and Tita) [9]. According to these authors, in these regions at least three sordida subcomplex species (*T. guasayana* and *T. sordida* group 1 and group 2) coexist in sympatry, together with the putative hybrids. Here, in Izozog we also identified three species (*T. guasayana*, *T. sordida sensu stricto* and *T. garciabesi*, respectively) and putative hybrids, similar to isoenzymes [9]. Since both heterochromatin and the ribosomal genes are inherited in Mendelian fashion [17, 21], the occurrence of heterozygous chromosomes for C-heterochromatin and the FISH patterns observed in the ribosomal cluster location (Fig. 2) suggests that these individuals are hybrids resulting from crosses among different taxa.

## Conclusions

By chromosomal markers, we recognize five chromosomal taxa and putative hybrids within the sordida subcomplex species in Argentina, Bolivia, Brazil and Paraguay. These morphologically similar taxa exhibit a striking karyological differentiation involving changes in the heterochromatin content and genome reorganization in the ribosomal clusters chromosomal position. In several regions, some of these taxa are sympatric and putative hybrids are detected. This paper identifies several erroneously classified populations, delimits the geographical distribution of each taxon and proposes the existence of a new cryptic species, widely distributed in Argentina. Most extensive population analyses (particularly from La Paz) and the application of other genetic techniques could resolve the taxonomic status of each chromosomal taxon. Considering the different epidemiological importance of these species, a morphological recognition key should be implemented for the selection of appropriate strategies for vector control.

## References

[1] Schofield CJ, Galvão C (2009). Classification, evolution and species groups within the Triatominae. Acta Trop..

[2] Lent H, Wygodzinsky P (1979). Revision of the Triatominae (Hemiptera, Reduviidae) and their significance as vector of Chagas disease. Bull Am Mus Nat Hist..

[3] Carcavallo RU, de Casas SI C, Sherlock IA, Galíndez-Girón I, Jurberg J, Galvão C, Carcavallo RU, Galíndez-Girón I, Jurberg J, Lent H (1999). Geographical distribution and alti-latitudinal dispersion. Chapter 17. Vol. III. Atlas of Chagas’ disease vectors in the Americas.

[4] Abalos JW, Wygodzinsky P (1951). Las Triatominae Argentinas (Reduviidae, Hemiptera). Inst Med Reg Public..

[5] Actis AS, Traversa OC, Carcavallo RU (1965). Estudios taxonómicos sobre el género *Triatoma* mediante la electroforesis de la linfa. An Esc Nac C Biol..

[6] Carcavallo RU, Cichero JA, Martínez A, Prosen AF, Ronderos R (1967). Una nueva especie del género *Triatoma* Laporte (Hemiptera, Reduviidae, Triatominae). 2as Jorn Entomoepidemiol Argentinas.

[7] Panzera F, Hornos S, Pereira J, Cestau R, Canale D, Diotaiuti L (1997). Genetic variability and geographic differentiation among three species of triatomine bugs (Hemiptera-Reduviidae). Am J Trop Med Hyg.

[8] Jurberg J, Galvão C, Lent H, Monteiro F, Macedo Lopes C, Panzera F (1998). Revalidação de *Triatoma garciabesi* Carcavallo, Cichero, Martínez, Prosen & Ronderos, 1967 (Hemiptera: Reduviidae). Entomol Vect.

[9] Noireau F, Gutierrez T, Zegarra M, Flores R, Brenière F, Cardozo L (1998). Cryptic speciation in *Triatoma sordida* (Hemiptera: Reduviidae) from the Bolivian Chaco. Trop Med Int Health..

[10] Noireau F, Gutierrez T, Flores R, Brenière F, Bosseno MF, Wisnivesky-Colli C (1999). Ecogenetics of *Triatoma sordida* and *Triatoma guasayana* (Hemiptera: Reduviidae) in the Bolivian Chaco. Mem Inst Oswaldo Cruz..

[11] Noireau F, Zegarra M, Ordoñez J, Gutierrez T, Dujardin JP (1999). Genetic structure of *Triatoma sordida* (Hemiptera: Reduviidae) domestic populations from Bolivia: Application on control interventions. Mem Inst Oswaldo Cruz..

[12] Gurgel-Gonçalves R, Ferreira JBC, Rosa AF, Bar ME, Galvão C (2011). Geometric morphometrics and ecological niche modelling for delimitation of near-sibling triatomine species. Med Vet Entomol..

[13] Calderón-Fernández GM, Juárez MP (2013). The cuticular hydrocarbons of the *Triatoma sordida* species subcomplex (Hemiptera: Reduviidae). Mem Inst Oswaldo Cruz..

[14] González-Britez NE, Carrasco HJ, Martínez Purroy CE, Feliciangeli MD, Maldonado M, López E (2014). Genetic and morphometric structures of *Triatoma sordida* (Hemiptera: Reduviidae) from the eastern and western regions of Paraguay. Front Public Health.

[15] Panzera F, Pérez R, Panzera Y, Ferrandis I, Ferreiro MJ, Calleros L (2010). Cytogenetics and genome evolution in the subfamily Triatominae (Hemiptera, Reduviidae). Cytogenet Genome Res..

[16] Panzera F, Ferreiro MJ, Pita S, Calleros L, Pérez R, Basmadjián Y (2014). Evolutionary and dispersal history of *Triatoma infestans*, main vector of Chagas disease, by chromosomal markers. Infect Genet Evol..

[17] Panzera Y, Pita S, Ferreiro MJ, Ferrandis I, Lages C, Pérez R (2012). High dynamics of rDNA cluster location in kissing bug holocentric chromosomes (Triatominae, Heteroptera). Cytogenet Genome Res..

[18] Pita S, Panzera F, Ferrandis I, Galvão C, Gómez-Palacio A, Panzera Y (2013). Chromosomal divergence and evolutionary inferences in Rhodniini based on the chromosomal location of ribosomal genes. Mem Inst Oswaldo Cruz..

[19] Schreiber G, Pellegrino J (1950). Eteropicnosi di autosomi come possibile meccanismo di speciazione. Sci Genet..

[20] Gorla DE, Jurberg J, Catalá SS, Schofield CJ (1993). Systematics of *Triatoma sordida*, *T. guasayana* and *T. patagonica* (Hemiptera, Reduviidae). Mem Inst Oswaldo Cruz.

[21] Panzera F, Dujardin JP, Nicolini P, Caraccio MN, Rose V, Tellez T (2004). Genomic changes of Chagas disease vector. South America. Emerg Infect Dis..

[22] Tamura K, Stecher G, Peterson D, Filipski A, Kumar S (2013). MEGA6: Molecular Evolutionary Genetics Analysis Version 6.0. Mol Biol Evol.

[23] García BA, Powell JR (1998). Phylogeny of species of *Triatoma* (Hemiptera: Reduviidae) based on mitochondrial DNA sequences. J Med Entomol..

[24] Hypsa V, Tietz DF, Zrzavý J, Rego RO, Galvão C, Jurberg J (2002). Phylogeny and biogeography of Triatominae (Hemiptera: Reduviidae): molecular evidence of a New World origin of the Asiatic clade. Mol Phylogenet Evol..

[25] Almeida CE, Marcet PL, Gumiel M, Takiya DM, Cardozo-de-Almeida M, Pacheco RS (2009). Phylogenetic and phenotypic relationships among *Triatoma carcavalloi* (Hemiptera: Reduviidae: Triatominae) and related species collected in domiciles in Rio Grande do Sul State. Brazil. J Vector Ecol..

[26] Justi SA, Russo CAM, Mallet JRS, Obara MT, Galvão C (2014). Molecular phylogeny of Triatomini (Hemiptera: Reduviidae: Triatominae). Parasites and Vectors.

[27] Rolón M, Vega MC, Román F, Gómez A, Rojas de Arias A (2011). First report of colonies of sylvatic *Triatoma infestans* (Hemiptera: Reduviidae) in the Paraguayan Chaco, using a trained dog. PLoS Negl Trop Dis.

[28] Gurgel-Gonçalves R, Galvão C, Costa J, Peterson AT. Geographic distribution of Chagas disease vectors in Brazil based on ecological niche modeling. J Tropical Med*.* 2012:705326. doi:10.1155/2012/705326. 10.1155/2012/705326 PMC3317230 22523500

[29] Diotaiuti L, Azeredo BVM, Busek SCU, Fernandes AJ (1998). Controle do *Triatoma sordida* no peridomicílio rural do município de Porteirinha, Minas Gerais. Brasil. Pan Am J Public Health..

[30] Noireau F, Brenière F, Ordoñez J, Cardozo L, Morochi W, Gutierrez T (1997). Low probability of transmission of *Trypanosoma cruzi* to humans by domiciliary *Triatoma sordida* in Bolivia. Trans R Soc Trop Med Hyg..

[31] Rossi JCN, Duarte EC, Gurgel-Gonçalves R (2015). Factors associated with the occurrence of *Triatoma sordida* (Hemiptera: Reduviidae) in rural localities of Central-West Brazil. Mem Inst Oswaldo Cruz..

[32] Monteiro FA, Donnelly MJ, Beard CB, Costa J (2004). Nested clade and phylogeographic analyses of the Chagas disease vector *Triatoma brasiliensis* in Northeast Brazil. Mol Phylogen Evol..

[33] Calleros L, Panzera F, Bargues MD, Monteiro FA, Klisiowicz DR, Zuriaga MA (2010). Systematics of *Mepraia* (Hemiptera–Reduviidae): cytogenetic and molecular variation. Infect Genet Evol. Infect Genet Evol..

[34] de la Rua N, Stevens L, Dorn PL (2011). High genetic diversity in a single population of *Triatoma sanguisuga* (LeConte, 1855) inferred from two mitochondrial markers: cytochrome b and 16S ribosomal DNA. Infect Genet Evol..

[35] Bar ME, Wisnivesky-Colli C (2001). *Triatoma sordida* Stål 1859 (Hemiptera, Reduviidae: Triatominae) in palms of Northeastern Argentina. Mem Inst Oswaldo Cruz..

[36] Bar ME, Damborsky MP, Oscherov EB, Milano AMF, Avalos G, Wisnivesky-Colli C (2002). Triatomines involved in domestic and wild *Trypanosoma cruzi* transmission in Concepción, Corrientes. Argentina. Mem Inst Oswaldo Cruz..

[37] Usinger RL, Wygodzinsky P, Ryckman RE (1966). The biosystematics of Triatominae. An Rev Entomol..

[38] Pietrokovsky S, Bottazi V, Gajate P, Canal D, Wisnivesky-Colli C (1994). Studies on reproductive compatibility between *Triatoma sordida* and *Triatoma guasayana*. Mem Inst Oswaldo Cruz..

[39] Rebagliati P, Papeschi AG, Mola LM, Pietrokovsky S, Gajate P, Bottazzi V (1998). Comparative meiotic studies in *Triatoma sordida* (Stål) and *T. guasayana* Wygodzinsky & Abalos (Reduviidae, Heteroptera). Mem Inst Oswaldo Cruz.

